# Self-efficacy in remitted bipolar disorder: associations with employment and self-esteem

**DOI:** 10.1590/1806-9282.20260500

**Published:** 2026-09-28

**Authors:** Esra Yalim, Cansu Unsal Mavi

**Affiliations:** 1 Çankırı State Hospital, Department of Psychiatry – Çankırı, Türkiye.; 2 Kartal Dr. Lütfi Kırdar City Hospital, Department of Psychiatry – Istanbul, Türkiye.

**Keywords:** Bipolar disorder, Employment, Self efficacy, Self esteem, Social support

## Abstract

**OBJECTIVE:**

The aim of this study was to examine general self-efficacy in relation to perceived social support, self-esteem, and employment status among individuals with remitted bipolar disorder.

**METHODS:**

This cross-sectional study included 67 patients with bipolar disorder in remission for at least eight weeks. Sociodemographic data were collected with a semi-structured form, and participants completed the Rosenberg Self-Esteem Scale, Multidimensional Scale of Perceived Social Support, and General Self-Efficacy Scale. Correlation analyses, group comparisons, and an exploratory multiple linear regression analysis were conducted.

**RESULTS:**

Self-esteem was positively correlated with perceived social support (r=0.390, p<0.01) and general self-efficacy (r=0.604, p<0.001). Employed participants had higher perceived social support, self-esteem, and general self-efficacy than unemployed participants (all p<0.05). In an exploratory multiple linear regression model, self-esteem and employment status remained associated with general self-efficacy after adjustment for perceived social support, age, and gender.

**CONCLUSION:**

Self-esteem and employment status were associated with general self-efficacy in patients with remitted bipolar disorder. These preliminary findings highlight the potential relevance of psychosocial and vocational factors, although longitudinal studies are needed to clarify the directionality of these relationships.

## INTRODUCTION

Bipolar disorder (BD) is a chronic psychiatric disorder characterized by recurrent episodes of mania and depression that adversely affect mood, cognition, and overall functioning^1^. Notably, psychosocial functional impairments may persist even during extended periods of euthymia^2^. BD is a major contributor to global disability, with unemployment rates consistently exceeding those observed in the general population^3^. Employment status has been associated with illness management and recovery trajectories in individuals with BD^4^. Accordingly, there is increasing recognition of the importance of identifying psychosocial factors that may support functional recovery in this population.

Self-esteem is defined as an individual's subjective evaluation of self-worth^5^. Individuals with BD have been shown to exhibit lower levels of self-esteem compared with healthy controls^6^. In addition, reduced perceived social support has been associated with greater symptom severity across psychiatric conditions, including BD^7^, and is also closely related to self-esteem^8^. Self-efficacy, conceptualized as the belief in one's capacity to initiate and sustain goal-directed behaviors, has been linked to quality of life in BD^9,10^. Higher self-efficacy has been associated with better psychological well-being and functional outcomes^11^. Both self-esteem and self-efficacy are well-established correlates of mental health outcomes^12^.

Self-esteem, perceived social support, and self-efficacy are distinct but interrelated psychosocial constructs relevant to functional recovery in BD. Although these constructs have been examined separately, their combined associations, particularly in relation to employment status, remain insufficiently explored. Therefore, this study aimed to examine the relationships among perceived social support, self-esteem, and general self-efficacy in individuals with remitted BD and to determine whether these variables differ according to employment status.

## METHODS

### Participants

A total of 67 patients diagnosed with BD according to DSM-5 criteria and considered clinically remitted were recruited from the psychiatry outpatient clinic of Çankırı State Hospital. Remission was determined by the treating psychiatrist based on clinical evaluation and was defined as the absence of acute mood symptoms and clinical stability for at least eight weeks^13^. Inclusion criteria comprised age between 18 and 65 years, a diagnosis of BD in remission, and willingness to participate in the study. Exclusion criteria included illiteracy, absence of remission, and the presence of systemic or neurological conditions that could potentially impair cognitive functioning.

### Data collection tools

Sociodemographic Data Form: A semi-structured questionnaire was used to obtain information on participants’ age, gender, education level, marital status, and psychiatric history.

Rosenberg Self-Esteem Scale (RSES): Self-esteem was assessed using the Rosenberg Self-Esteem Scale developed by Rosenberg and adapted into Turkish by Çuhadaroğlu. The instrument comprises 10 items rated on a 4-point Likert scale and includes both positive and negative self-esteem subdimensions. Total scores range from 0 to 30, with higher scores indicating higher levels of self-esteem^14,15^.

Multidimensional Scale of Perceived Social Support (MSPSS): Perceived social support was evaluated using the MSPSS developed by Zimet et al. and validated in Turkish by Eker et al. This 12-item self-report instrument includes three subscales: family, friends, and significant other. Items are rated on a 7-point Likert scale, with higher scores indicating greater perceived social support^16,17^.

General Self-Efficacy Scale (GSES): General self-efficacy was assessed using the scale developed by Sherer et al. and validated in Turkish by Yildirim and Ilhan. The 17-item version of this self-report instrument was used in the present study. Items are rated on a 5-point Likert scale, and higher total scores indicate greater self-efficacy^18,19^.

### Procedure

Ethical approval was obtained from the Çankırı Karatekin University Scientific Research Ethics Committee (14/07/2025–22). Written informed consent was obtained from all participants prior to study inclusion. The study was conducted in accordance with the principles of the Declaration of Helsinki.

### Statistical analysis

Data were analyzed using SPSS version 22.0. Descriptive statistics were reported as mean±standard deviation or n (%). Pearson correlation analyses were used to examine bivariate associations, and independent-samples t-tests were used for comparisons by employment status. An exploratory multiple linear regression analysis was conducted to examine variables associated with general self-efficacy. Education level was not entered into the final regression model because of its limited variability in the present sample and to avoid overfitting in the context of the modest sample size. Statistical significance was set at p<0.05. A sample size calculation using G*Power 3.1 indicated that approximately 70 participants would be required for a multiple linear regression model with five predictors, assuming a medium effect size close to Cohen's f^2^=0.20, α=0.05, and power=0.80.

## RESULTS

### Sample characteristics

The study sample comprised 67 patients with BD. The mean age was 32.26 years (SD=9.15). The majority of participants were female (77.6%, n=52), single or separated (67.2%, n=45), and had attained a university-level education or higher (79.1%, n=53). Slightly more than half of the participants were employed (52.2%, n=35).

### Descriptive statistics

The mean total perceived social support score was 55.09 (SD=21.98), the mean self-esteem score was 25.52 (SD=6.85), and the mean general self-efficacy score was 53.40 (SD=13.94).

### Correlation analyses

Pearson correlation analyses indicated that self-esteem was positively correlated with total perceived social support (r=0.390, p<0.01). General self-efficacy was correlated with self-esteem (r=0.604, p<0.001) and demonstrated weaker but statistically significant correlations with friend support (r=0.278, p<0.05) and significant other support (r=0.251, p<0.05), whereas no significant correlation was observed with family support or total perceived social support. Detailed correlation coefficients are presented in Table 1.

**Table 1:** Correlations between perceived social support, self-esteem, and general self-efficacy.

	Family support	Friend support	Significant other support	MSPSS	RSES	GSES
Family support	–					
Friend support	0.592***	–				
Significant other support	0.539***	0.717***	–			
MSPSS	0.806***	0.897***	0.884***	–		
RSES	0.237	0.433***	0.330**	0.390**	–	
GSES	0.059	0.278*	0.251*	0.233	0.604***	–

GSES: General Self-Efficacy Scale; MSPSS: Multidimensional Scale of Perceived Social Support; RSES: Rosenberg Self-Esteem Scale.

* p<0.05,

** p<0.01,

*** p<0.001.

### Group comparisons by employment status

Compared with unemployed participants, employed participants reported significantly higher total perceived social support scores [MSPSS: t(65)=-3.02, p=0.004], self-esteem scores [RSES: t(65)=-4.62, p<0.001], and general self-efficacy scores [GSES: t(65)=-4.59, p<0.001]. Detailed group comparisons are presented in Table 2.

**Table 2:** Comparison of social support, self-esteem, and self-efficacy by employment status in bipolar disorder (n=67).

Variable	Unemployed (mean±SD)	Employed (mean±SD)	t	df	p
MSPSS	47.77±22.61	63.09±18.48	-3.02	65	0.004
RSES	22.29±6.52	29.06±5.36	-4.62	65	<0.001
GSES	46.86±13.02	60.56±11.25	-4.59	65	<0.001

GSES: General Self-Efficacy Scale, MSPSS: Multidimensional Scale of Perceived Social Support, RSES: Rosenberg Self-Esteem Scale; SD: standard deviation.

### Exploratory multiple linear regression analysis

The exploratory regression model was significant overall [R^2^=0.420, adjusted R^2^=0.372, F(5,61)=8.82, p<0.001]. Self-esteem was positively associated with general self-efficacy (B=1.001, SE=0.243, p<0.001), and employed participants had higher general self-efficacy scores than unemployed participants when all variables were entered simultaneously (B=7.576, SE=3.326, p=0.026). Variance inflation factor (VIF) values ranged from 1.044 to 1.514, indicating no evidence of problematic multicollinearity. Detailed regression analysis results are presented in Table 3.

**Table 3:** Exploratory multiple linear regression model (n=67).

Variable	B	SE	LLCI	ULCI	t	p	Standardized estimate (β)
Intercept	26.432	7.386	11.662	41.201	3.579	p<0.001	
RSES	1.001	0.243	0.516	1.487	4.123	p<0.001	0.493
MSPSS	-0.029	0.072	-0.173	0.114	-0.408	p=0.685	-0.046
Age	-0.029	0.166	-0.360	0.302	-0.177	p=0.860	-0.019
Gender (female vs. male)	1.596	3.309	-5.021	8.212	0.482	p=0.631	0.114
Employment status (employed vs. unemployed)	7.576	3.326	0.926	14.226	2.278	p=0.026	0.543

MSPSS: Multidimensional Scale of Perceived Social Support; RSES: Rosenberg Self-Esteem Scale; β: standardized regression coefficient; CI: 95% confidence interval; LLCI: lower limit of confidence interval; ULCI: upper limit of confidence interval; SE: standard error; Gender was coded as Female=1, Male=0. Employment status was coded as employed=1, unemployed=0.

## DISCUSSION

In this study, self-esteem was positively associated with both perceived social support and general self-efficacy in patients with BD in clinical remission. Employed participants reported higher levels of these psychosocial variables than unemployed participants. In the exploratory adjusted model, self-esteem and employment status remained associated with general self-efficacy.

Consistent with previous evidence^8,20^, the present study found a positive association between perceived social support and self-esteem. These findings suggest that social support and self-esteem are interrelated psychosocial constructs in BD.

Self-efficacy has been recognized as an important correlate of mental health outcomes^21^, and previous clinical research has reported positive associations among self-efficacy, self-esteem, and social support^11^. In the present study, self-esteem remained associated with general self-efficacy in the exploratory regression model. This finding is consistent with evidence that self-esteem and self-efficacy are closely related dimensions of self-perception^20^ and may reflect the link between self-evaluation and perceived capability. However, it should be interpreted cautiously because the two constructs may share psychological variance.

General self-efficacy was not significantly associated with total perceived social support; only friend support and significant-other support showed small associations, whereas family support was not significant. This pattern may suggest that peer and significant-other support are more closely related to perceived competence than family support in this adult sample, although this interpretation remains tentative. Given the content of the GSES, which assesses perceived competence, initiative, persistence, and coping ability^18^, these exploratory subscale findings may reflect the relevance of specific interpersonal sources of support rather than overall perceived support. However, they should not be interpreted as evidence of a robust or mediated relationship between social support and self-efficacy.

Employment has been associated with psychosocial functioning in BD^22^. In the present study, employed participants reported higher self-esteem, self-efficacy, and perceived social support than unemployed participants, and employment status remained associated with general self-efficacy in the exploratory regression model. This finding is consistent with evidence linking employment variables to self-esteem in severe mental illness^23^ and with studies showing associations between self-efficacy and functional impairment in schizophrenia^24^. However, given the cross-sectional design, the absence of data on premorbid employment status and current subthreshold symptoms, and the relatively homogeneous sample, the directionality of this association remains uncertain. Thus, employment status should be regarded as a contextual correlate rather than a causal factor, and the magnitude of this association should be interpreted cautiously.

### Limitations

This study has several limitations. Its cross-sectional design precludes causal inference, and the observed associations, particularly between employment status and self-efficacy, may be bidirectional. The relatively small, single-center sample limits generalizability and may have reduced the power to detect small effects, especially in subgroup analyses; therefore, non-significant findings should be interpreted with caution. In addition, recruitment from a single center and participation on a voluntary basis may have introduced selection bias. Reliance on self-report measures may have introduced response bias. The predominance of female and highly educated participants may also have resulted in a more homogeneous and higher-functioning sample, potentially influencing regression estimates and limiting the applicability of the findings to more diverse clinical populations. Moreover, the relatively wide confidence interval for the employment-status coefficient suggests imprecision in the estimated magnitude of this association, and the corresponding regression estimate should therefore be interpreted cautiously. Several relevant confounders, including illness duration, number of previous episodes, premorbid employment history, current subthreshold mood symptoms, and psychotropic medication use, were not assessed. Remission status was determined clinically without standardized clinician-rated mood scales, which limits the objectivity of remission assessment. Finally, variables such as self-esteem and self-efficacy may fluctuate over time, which could not be captured in the present design.

## CONCLUSION

In conclusion, self-esteem was positively associated with both perceived social support and general self-efficacy in patients with clinically remitted BD. In an exploratory adjusted model, self-esteem and employment status remained associated with general self-efficacy. Given the cross-sectional design, modest sample size, and clinically determined remission status, these findings should be interpreted cautiously, and longitudinal studies with standardized mood assessments are needed to clarify directionality.

## Data Availability

The datasets generated and/or analyzed during the current study are available from the corresponding author upon reasonable request.
